# Antioxidant and Antimicrobial Attributes and Phenolics of Different Solvent Extracts from Leaves, Flowers and Bark of Gold Mohar [*Delonix regia* (Bojer ex Hook.) Raf.] 

**DOI:** 10.3390/molecules16097302

**Published:** 2011-08-25

**Authors:** Ghulam Shabir, Farooq Anwar, Bushra Sultana, Zafar M. Khalid, Muhammad Afzal, Qaiser M. Khan, M. Ashrafuzzaman

**Affiliations:** 1 Department of Chemistry and Biochemistry, University of Agriculture, Faisalabad 38040, Pakistan; 2 Environmental Biotechnology Division, National Institute for Biotechnology and Genetic Engineering (NIBGE), P.O.Box 577, Jhang Road, Faisalabad, Pakistan; 3 Institute of Tropical Agriculture, Universiti Putra Malaysia, 43400 UPM, Serdang, Selangor Malaysia

**Keywords:** *Delonix regia*, solvent extracts, total phenolics, antioxidant activities, antimicrobial activities

## Abstract

This paper describes the antioxidant and antimicrobial activities and phenolic components of different solvent (absolute methanol, absolute ethanol, absolute acetone, 80% methanol, 80% ethanol, 80% acetone and deionized water) extracts of leaves, flowers and bark of Gold Mohar [*Delonix regia* (Bojer ex Hook.) Raf.]. The extract yields from leaves, flowers and bark ranged from 10.19 to 36.24, 12.97 to 48.47 and 4.22 to 8.48 g/100 g dry weight (DW), respectively. Overall, 80% methanol extract produced from the leaves exhibited significantly (P < 0.05) higher antioxidant activity, with high phenolic contents (3.63 g GAE/100 g DW), total flavonoid contents (1.19 g CE/100 g DW), inhibition of peroxidation (85.54%), DPPH scavenging capacity (IC_50_ value 8.89 μg/mL) and reducing power (1.87). Similarly, this 80% methanol leaves extract also showed superior antimicrobial activity. HPLC analysis of the 80% methanol extracts for individual phenolics revealed the presence of gallic, protocatechuic and salicylic acid in leaves; gallic, protocatechuic, salicylic, *trans*-cinnamic and chlorogenic acid in flowers, and gallic acid in bark as the main (amount > 1.50 mg/100 g DW) phenolic acids. Besides, small amounts (<1.50 mg/100 g DW) of some other phenolic acids such as sorbic, sinapic, *p*-coumaric, *m*-coumaric, ferulic, caffeic, 3-hydroxybenzoic, 4-hydroxycinnamic and 4-hydroxybenzoic acids were also detected. The extracts of the tested parts of Gold Mohar, especially, the leaves, might be valuable for functional food and therapeutic applications.

## 1. Introduction

Gold Mohar [*Delonix regia* (Bojer ex Hook.) Raf.] also known as Royal Poinciana, Flamboyant and Flame tree is a species of flowering plant, belonging to the family *Caesalpiniaceae*. This tree, a native of Madagascar, but now distributed in several countries of the tropical region, is often used to prepare extracts with antimicrobial and antifungal activities [1,2,3]. Gold Mohar, with an impressive range of medicinal and biological properties, has been used in the folk medicine systems of several civilizations for the treatment of constipation, inflammation, arthritis, hemiplagia, leucorrhoea and rheumatism [4,5].

Reactive oxygen species (ROS) such as hydroxyl radicals, peroxides and superoxide anions are one of the major causes of oxidative damage causing carcinogenesis, mutagenesis, aging and cardiovascular diseases [6,7]. Antioxidants inhibit or delay the oxidation process by blocking the initiation or propagation of oxidizing chain reactions and hence protect the human body from oxidative stress-related diseases [8]. They are also used to protect foods from rancidity, discoloration or deterioration due to auto-oxidation [9]. Though synthetic antioxidants such as butylated hydroxyanisole, butylated hydroxytoluene and propyl gallate are added to food products to retard lipid oxidation, there are restrictions on their use because of the negative perception of consumers due to their perceived carcinogenic potential [10]. On the other hand, owing to their multiple health benefits, the demand for plant-derived natural antioxidants has increased greatly [9,10,11].

In recent years, a huge number of medicinal and food plants have attracted a great deal of scientific and public interest with regard to their potential uses in folk medicine and as sources of natural antioxidant and antimicrobial agents [11,12,13]. Consequently, the antioxidant and antimicrobial activities of plant extracts have formed the basis of many applications, including food preservation, pharmaceuticals, functional foods and nutraceuticals, and alternative medicinal therapies [14,15,16].

It is well established that phenolic antioxidants, namely flavonoids and phenolic acids, are commonly distributed in plant leaves, flowering tissues and woody parts such as stem and bark. In plants, these antioxidant phenolics play a vital role for normal growth and protection against infection and injuries from internal and external sources [17,18].

Although some earlier studies revealed the medicinal attributes of Gold Mohar however, very little work has been done on the comparative research about the detailed antioxidant and antimicrobial properties of leaves, flowers and bark of this plant indigenous to sub-continental region and especially from Pakistan, so the present study was undertaken with the main objective of evaluating their antioxidant and antimicrobial activities and to quantify phenolic acids in different parts of this valuable tree.

## 2. Results and Discussion

### 2.1. Extraction Yields

**Figure 1:**
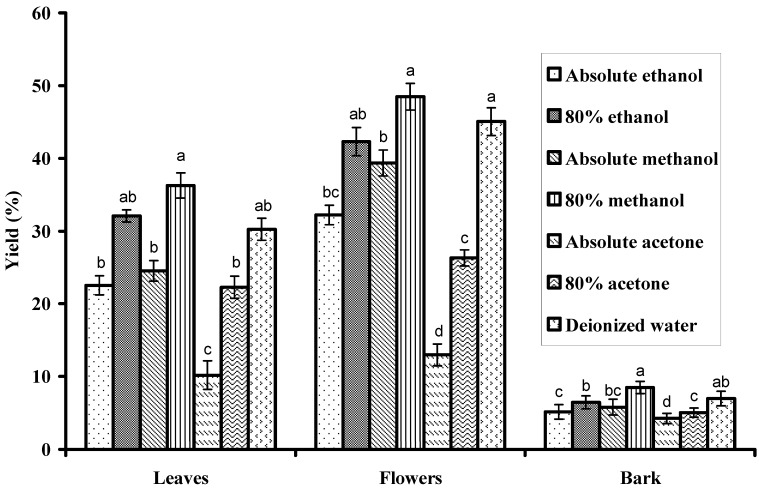
Yield (g/100 g DW) from different parts of Gold Mohar. Values are mean ± SD (n = 3 × 3) of triplicates samples. Different superscript letters on bars indicate significant (P < 0.05) differences of means among the extracting solvents.

The extraction yields from leaves, flowers and bark of Gold Mohar (*Delonix regia*) using different solvents are presented in Figure 1. Comparatively, 80% methanol exhibited higher extraction yields from flowers (48.5%) and leaves (36.24%) than from bark (8.5%). The extraction ability of different solvents for recovering extractable components from leaves followed the order: 80% methanol > 80% ethanol > deionized water > absolute methanol > absolute ethanol > 80% acetone > absolute acetone. Yields of extract from flowers and bark followed the same order: 80% methanol > deionized water > 80% ethanol > absolute methanol > absolute ethanol > 80% acetone > absolute acetone. The higher extract yields obtained in the present study with 80% methanol were in good agreement with the previous findings of Siddhuraju and Becker [19], who reported the highest extract yield from *Moringa oleifera* Lam. leaves using 80% methanol. Likewise, Sultana *et al.* [20] also reported that 80% methanol provided the maximum extract yields from different medicinal plant parts. In a previous study Aqil and Ahmad [21] reported a maximum yield (15%) of Gold Mohar flowers extract with 70% methanol, however we did not find any report on the extraction yields from leaves and barks to compare with our present data. Significant (P < 0.05) differences of extract yields among different solvents and plant parts might be attributed to the varied polarity of the solvents used as well as the availability of different extractable components in each part of plant [20].

### 2.2. Total Phenolics and Flavonoids Contents

The total phenolic contents (TPC) and total flavonoid contents (TFC) determined in leaves, flowers and bark extracts of Gold Mohar are shown in Table 1. The TPC and TFC of the leaves extracts varied significantly (P < 0.05), ranging from 0.89–3.63 g GAE/100 g DW and 0.17–1.19 g CE/100 g DW, respectively. Among different solvent extracts tested in this study, 80% methanol extract of leaves showed the highest TPC and TFC, followed by flowers and bark, respectively. 80% methanol is known to be an efficient and widely used solvent to extract phenolics and flavonoids from plant materials, due to the fact that methanol-water mixtures are highly polar and thus show greater efficacy in the extraction of polar phytochemicals such as phenolics and flavonoids [22].

The extracted amounts of total phenolics and total flavonoids from flowers in the present study were higher than those reported previously from Gold Mohar flowers [5,21]. However, the present levels of TPC and TFC in the bark were lower than those previously reported for Gold Mohar bark [23]. The presence of phenolics and flavonoids is affected by the type of plant parts, maturity at harvest, growing conditions, soil conditions and post-harvest treatment [24]. Different parts of the same plant may synthesize and accumulate different compounds or different amounts of a particular compound due to their differential gene expression, which in turn affects the antioxidant activity and other biological properties of the plant extracts produced [25,26]. Many studies have confirmed that the amounts and composition of phenolic and flavonoid compounds is diversified at the sub-cellular level and within plant tissues as well [27,28].

### 2.3. HPLC Analysis of Phenolic Compounds

Among the tested solvents, 80% methanol was found to be the most efficient solvent to extract TPC from leaves, flowers and bark of Gold Mohar. So this solvent extract was further analyzed by HPLC to quantify the individual phenolic acids in the selected parts of the plant (Table 2). Of the thirty phenolic compounds tested by HPLC only fifteen were detected in leaves, flowers and bark of Gold Mohar. These compounds showed significant variations (P < 0.05) among plant parts. Gallic acid, salicylic acid and protocatechuic acid were the most abundant phenolic acids in the leaves. Others, such as protocatechuic, *trans*-cinnamic, salicylic, chlorogenic and gallic acid were found to be the major phenolics in flowers, while, gentisic and gallic acids were the main phenolic components in the bark of Gold Mohar. Adje *et al*. [3] reported the presence of gallic acid and protocatechuic acid in the flowers extract of Gold Mohar but they did not find other phenolics in their study that were detected in the present work, *i.e.*, caffeic acid, salicylic acid, ferulic acid, gentisic acid, chlorogenic acid, 3-hydroxybenzoic acid, 4-hydroxycinnamic acid, and 4-hydroxybenzoic acid. All of the detected phenolic compounds are known to have antioxidant properties [11,29,30,31]. Gallic acid, which is efficiently absorbed in human body, shows positive effects against cancer cell under *in vitro* condition [32]. *p*-coumaric acid is believed to reduce the risk of stomach cancer by reducing the formation of carcinogenic nitrosamines [33].

**Table 1:** Antioxidant activity of Gold Mohar leaves, flowers and bark extracts with different solvents.

Parameters	Solvent extracts						
*Leaves*	Absolute	80%	Absolute	80%	Absolute	80%	Deionized
	Etanol	ethanol	methanol	methanol	acetone	acetone	water
TPC (g/100 g DW)	1.48 ± 0.06 ^bc^	2.31 ± 0.08 ^b^	2.40 ± 0.07 ^b^	3.63 ± 0.12 ^a^	1.01 ± 0.02 ^c^	1.21 ± 0.05 ^c^	0.89 ± 0.04 ^d^
TFC (g/100 g DW)	0.52 ± 0.03 ^bc^	0.78 ± 0.02 ^b^	0.99 ± 0.05 ^ab^	1.19 ± 0.07 ^a^	0.27 ± 0.02 ^d^	0.42 ± 0.01 ^c^	0.17 ± 0.03 ^cd^
DPPH, IC_50_ (μg/mL)	18.78 ± 0.5 ^b^	16.53 ± 0.7 ^bc^	13.26 ± 0.4 ^bc^	8.89 ± 0.46 ^c^	24.11 ± 0.81 ^ab^	22.74 ± 0.72 ^b^	34.93 ± 0.92 ^a^
Inhibition of linoleic acid peroxidation (%)	68.08 ± 2.8 ^b^	74.02 ± 3.5 ^ab^	79.58 ± 3.1 ^b^	85.54 ± 4.1 ^a^	48.14 ± 2.3 ^c^	52.56 ± 2.1 ^b^	32.26 ± 1.6 ^ab^
***Flowers***							
TPC (g/100 g DW)	1.28 ± 0.04 ^cd^	1.36 ± 0.06 ^bc^	1.68 ± 0.08 ^b^	2.24 ± 0.11 ^a^	0.36 ± 0.02 ^d^	0.42 ± 0.05 ^cd^	1.02 ± 0.02 ^c^
TFC (g/100 g DW)	0.41 ± 0.02 ^bc^	0.47 ± 0.02 ^b^	0.48 ± 0.02 ^b^	0.81 ± 0.03 ^a^	0.11 ± 0.01 ^d^	0.27 ± 0.03 ^c^	0.31 ± 0.04 ^bc^
DPPH, IC_50_ (μg/mL)	26.61 ± 1.1 ^ab^	22.68 ± 1.02 ^b^	16.66 ± 0.39 ^c^	14.80 ± 0.48 ^c^	44.58 ± 2.3 ^a^	38.02 ± 0.9 ^ab^	31.74 ± 0.8 ^b^
Inhibition of linoleic acid peroxidation (%)	57.62 ± 2.3 ^bc^	61.15 ± 2.8 ^ab^	73.43 ± 3.2 ^b^	79.69 ± 3.7 ^a^	40.01 ± 1.8^ d^	42.48 ± 2.3 ^c^	45.78 ± 1.8 ^a^
***Bark***							
TPC (g/100 g DW)	0.42 ± 0.02 ^c^	0.49 ± 0.01 ^b^	0.58 ± 0.01 ^bc^	0.69 ± 0.02 ^a^	0.10 ± 0.01 ^d^	0.16 ± 0.01 ^cd^	0.09 ± 0.01 ^c^
TFC (g/100 g DW)	0.16 ± 0.01 ^b^	0.18 ± 0.01 ^c^	0.21 ± 0.01 ^ab^	0.28 ± 0.02 ^a^	0.09 ± 0.00 ^d^	0.18 ± 0.01 ^cd^	0.07 ± 0.01 ^c^
DPPH, IC_50_ (μg/mL)	41.45 ± 1.8 ^b^	36.16 ± 1.3 ^c^	34.14 ± 1.8 ^c^	29.86 ± 1.2 ^d^	44.64 ± 1.8 ^b^	49.98 ± 1.9 ^ab^	58.84 ± 1.8 ^a^
Inhibition of linoleic acid peroxidation (%)	32.23 ± 1.2 ^c^	38.35 ± 2.3 ^b^	42.1 ± 2.6 ^bc^	52.3 ± 2.3 ^a^	22.88 ± 1.9 ^d^	25.12 ± 1.2 ^c^	19.87 ± 1.2 ^d^

TPC: Total phenolic contents expressed as gallic acid equivalent; TFC: Total flavonoid contents expressed as catechin equivalent; Values are mean ± SD (n = 3 × 3) of three separate experiments. Different superscript letters within the same row indicate significant (P < 0.05) differences of means among the extracting solvents.

**Table 2:** HPLC quantification of 80% methanol soluble phenolic acids (mg/100 g DW) identified in different parts of Gold Mohar.

Compounds	Leaves	Flowers	Bark
Sorbic acid	1.02 ± 0.06 ^a^	0.14 ± 0.02 ^b^	ND
Sinapic acid	1.21 ± 0.03 ^a^	0.80 ± 0.05 ^b^	0.95 ± 0.03 ^b^
*p*-Coumaric acid	1.24 ± 0.07 ^a^	1.26 ± 0.05 ^a^	0.15 ± 0.01 ^b^
Protocatechuic acid	5.81 ± 0.32 ^a^	4.24 ± 0.26 ^a^	ND
*m*-Coumaric acid	0.57 ± 0.02 ^a^	0.16 ± 0.01 ^b^	ND
*trans*-Cinnamic acid	ND	3.95 ± 0.16 ^a^	0.46 ± 0.02 ^b^
Ferulic acid	1.12 ± 0.05 ^a^	0.47 ± 0.03 ^b^	ND
Caffeic acid	1.29 ± 0.06 ^a^	0.10 ± 0.01 ^b^	ND
Salicylic acid	5.43 ± 0.28 ^a^	4.01 ± 0.16 ^a^	ND
Gentisic acid	ND	1.45 ± 0.04 ^a^	1.20 ± 0.05 ^b^
Chlorogenic acid	1.53 ± 0.06 ^a^	2.13 ± 0.03 ^b^	ND
Gallic acid	8.15 ± 0.46 ^a^	6.16 ± 0.41 ^b^	1.99 ± 0.06 ^c^
3-Hydroxybenzoic acid	0.76 ± 0.03 ^b^	0.98 ± 0.04 ^a^	ND
4-Hydroxycinnamic acid	ND	0.18 ± 0.01	ND
4-Hydroxybenzoic acid	ND	1.09 ± 0.05 ^a^	0.91 ± 0.01 ^b^

ND: not detected. Values are mean ± SD (n = 3 × 3) of three separate experiments. Different superscript letters within the same row indicate significant (P < 0.05) differences of means within the plant parts.

### 2.4. Reducing Power of Extracts

Different studies have indicated that the antioxidant potential of certain compounds is related to their reducing power [34,35], thus reducing power assays may serve as an important indicator of prospective antioxidant activity in a plant extract. In this assay, ferric ions (Fe^3+^) are reduced to ferrous ions (Fe^2+^) and with it change in color from yellow to bluish green. The intensity of color depends on the reducing potential of the compounds present in the media. Greater the intensity of the color, greater will be the absorption; consequently, greater will be the antioxidant activity [36].

**Figure 2:**
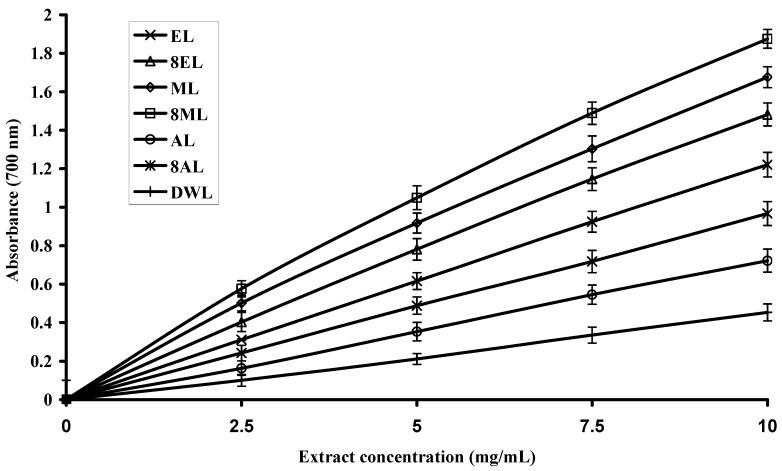
Reducing power of extracts from leaves of Gold Mohar. Each value represents mean ± SD (n = 3 × 3). EL: Ethanol leaves extract; 8EL: 80% Ethanol leaves extract; ML: Methanol leaves extract; 8ML: 80% Methanol leaves extract; AL: Acetone leaves extract; 8AL: 80% Acetone leaves extract; DWL: Deionized water leaves extract.

**Figure 3:**
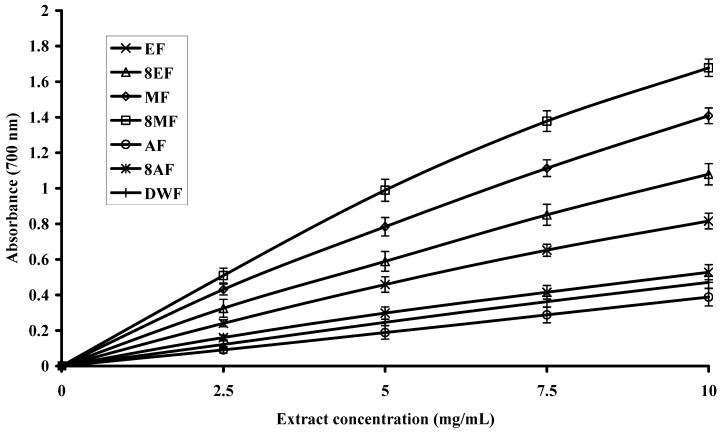
Reducing power of extracts from flowers of Gold Mohar. Each value represents mean ± SD (n = 3 × 3). EF: Ethanol flowers extract; 8EF: 80% Ethanol flowers extract; MF: Methanol flowers extract; 8MF: 80% Methanol flowers extract; AF: Acetone flowers extract; 8AF: 80% Acetone flowers extract; DWF: Deionized water flowers extract.

**Figure 4:**
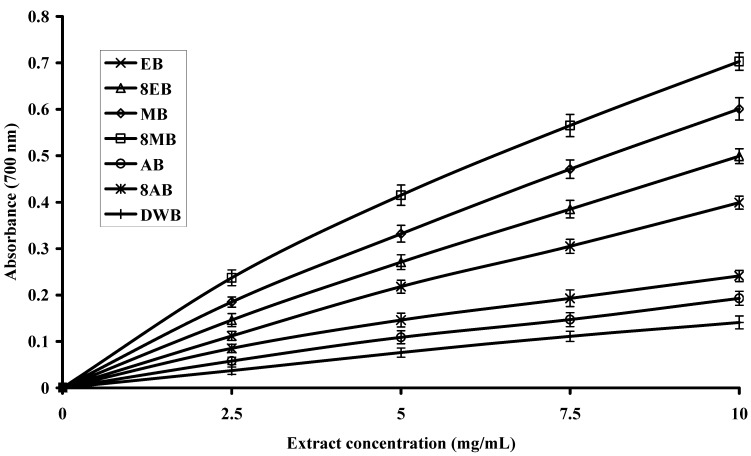
Reducing power of extracts from bark of Gold Mohar. Each value represents mean ± SD (n = 3 × 3). EB: Ethanol bark extract; 8EB: 80% Ethanol bark extract; MB: Methanol bark extract; 8MB: 80% Methanol bark extract; AB: Acetone bark extract; 8AB: 80% Acetone bark extract; DWB: Deionized water bark extract.

The trends of reducing potential for leaves, flowers and bark extracts are presented in Figure 2, Figure 3 and Figure 4, respectively. The reducing potential of the tested extracts observed over a concentration range of 2.5 to 10.0 mg/mL, showed a general increase in activity when the concentration was increased. Relatively, the extracts from leaves and flowers showed significantly (P < 0.05) higher reducing power than bark, regardless of the solvent used. However, 80% methanol extract from leaves had the highest reducing power (absorbance value = 1.875 at 10 mg/mL, Figure 2). The 80% methanol extract from flowers showed a moderate reducing power (absorbance value = 1.678 at 10 mg/mL, Figure 3), while 80% methanol extract from bark exhibited only a slight reducing power (absorbance value = 0.703 at 10 mg/mL, Figure 4). The variation in the reducing powers of different solvent extracts was statistically significant (P < 0.05). The reducing power of extracts of Gold Mohar determined in this work was noted to be lower than that seen for extracts of *Lippia alba* by Ara and Nur [17]. However, our results are comparable to the previous findings of Sultana *et al*. [20] who reported the reducing power of 80% methanol extracts from leaves of *Moringa oleifera* Lam. and bark of *Acacia nilotica,* at concentrations of 10 mg/mL, to be 1.78 and 1.13, respectively.

### 2.5. Antioxidant Activity of Extracts in Linoleic Acid Peroxidation System

The antioxidant activity of extracts has also been evaluated by measuring inhibition of peroxidation in linoleic acid system using the thiocyanate method [37]. Table 1 shows the percentage inhibition of linoleic acid peroxidation for extracts of different parts of Gold Mohar. Inhibition of peroxidation in leaves (85.54%) and flowers (79.69%) extracts (80% methanol) was significantly (P < 0.05) higher than that of bark extract (52.3%), while butylated hydroxytoluene (BHT) and ascorbic acid, used as positive controls for comparison purposes, offered 85.11% and 49.28%, inhibition of peroxidation, respectively. Overall, 80% methanolic extract of leaves was found to be comparable with BHT. On the other hand, when compared to ascorbic acid, all the tested leaves extracts were found to have significantly (P < 0.05) higher inhibition of peroxidation. This higher inhibition of peroxidation exhibited by leaves extract might be due to the presence of greater amounts of phenolics and flavonoids in the leaves [38]. The inhibition of peroxidation of the extracts from Gold Mohar was found to be higher than that reported for *Cassia fistula* by Siddhuraju *et al.* [35]. However, present values for inhibition of peroxidation were comparable to those of *Azadirachta indica, Terminalia arjuna* and *Acacia nilotica* [39].

### 2.6. DPPH Radical Scavenging Assay

The DPPH radical scavenging assay has been widely used to assess the antioxidant ability of various plants extracts and natural products [39,40]. This method is based on the ability of DPPH radical to react with hydrogen donor species such as phenolics and flavonoids present in the extract material. Upon receiving a proton from the donor species it loses its color and becomes yellow. As the concentration of phenolic compounds increases their DPPH radical scavenging activity also increases [41].

The free radical scavenging activity of leaves, flowers and bark extract of Gold Mohar was investigated by DPPH^•^ assay (Table 1). The free radical scavenging capacity was increased with increasing extract concentration, especially, the leaves extract followed by flowers extract had stronger DPPH^•^ radical scavenging activity. IC_50_ (the extract concentration inhibiting 50% of DPPH radicals) values for leaves and flowers extracts ranged from 8.89 to 34.93 and 14.80 to 44.58 μg/mL, respectively. However, poorer antioxidant capacity was exhibited by bark extract, with IC_50_ values 29.86 to 58.84 μg/mL. As expected, 80% methanol extracts exhibited superior scavenging activity than other solvent extracts. The results for superior DPPH radical scavenging capacity of the leaves and flowers extracts could be explained by the presence of greater concentration of phenolics in leaves and flowers [35]. DPPH radical scavenging activity of Gold Mohar was found to be in consistent with previous studies [1,20].

### 2.7. Comparison between Different Antioxidant Assays

The assessment of antioxidant activity of plant materials using different methods is widely accepted. Taking into account the results of the different antioxidant assays such as inhibition of lipid peroxidation, DPPH radical scavenging capacity, measurement of reducing potential combined with the estimation of total phenolic and total flavonoid contents were compared and correlated with each other (Table 3). A good correlation (r = 0.988) between TPC and TFC was observed. The correlation between TPC and TFC has been widely studied in different food stuffs such as fruit, vegetables and plant material [11,42,43]. A stronger positive correlation (r = 0.933) between TPC and reducing power of extracts suggested that phenolic compounds might have acted as powerful reducing agent in Gold Mohar. Li *et al.* [44,45] have reported the existence of similar linear relationships between reducing power and TPC. Similarly, a high correlation (r = 0.975) was exhibited between TFC and reducing power. This is in agreement with the study of Sultana *et al*. [39] who also observed a strong positive correlation between TFC and reducing power during antioxidant activity evaluation of different bark extracts. These results support the basic concept that antioxidants are reducing agents. High correlation (r = 0.937) was also observed between TFC and % inhibition of peroxidation data. Furthermore, positive correlation (r = 0.883) was observed between TPC and % inhibition. This might be ascribed to the fact that leaves, flowers and bark extracts of Gold Mohar showing greater amount of TPC and TFC also exhibited higher levels of percent inhibition of linoleic acid peroxidation. These results are in line with the findings of Anwar *et al.* [46] who reported that extracts with higher TPC also showed strong activity against linoleic acid peroxidation. DPPH^•^ exhibited negative linear correlation with TPC (r = −0.959), TFC (r= −0.985), % inhibition (r = −0.977) and reducing power (r = −0.984). This shows that IC_50_ values are inversely proportional to TPC, TFC, % inhibition and reducing power. Consequently, DPPH radical scavenging activity is strongly associated with phenolic and flavonoid compounds in this plant. A similar observation as our present has been reported by Sultana *et al.* [39].

Furthermore, variation in correlation coefficient among different antioxidant assays indicates that a single assay is not sufficient to evaluate the total antioxidant activity of a specific plant material [47,48].

**Table 3:** Comparison between different antioxidant assays as represent by correlation coefficient (r).

Variable	TPC	TFC	DPPH	% Inhibition	RP
**TPC**	-	0.988	−0.959	0.883	0.933
		*P* = 0.000	*P* = 0.000	*P* = 0.000	*P* = 0.000
**TFC**	0.988	-	−0.985	0.937	0.975
	*P* = 0.000		*P* = 0.000	*P* = 0.000	*P* = 0.000
**DPPH**	−0.959	−0.985	-	−0.977	−0.984
	*P *= 0.000	*P* = 0.000		*P* = 0.000	*P* = 0.000
**% Inhibition**	0.883	0.937	−0.977	-	0.980
	*P *= 0.000	*P *= 0.000	*P* = 0.000		*P* = 0.000
**RP**	0.933	0.975	−0.984	0.980	-
	*P* = 0.000	*P* = 0.000	*P* = 0.000	*P* = 0.000	

Correlation analysis between different antioxidant assays (n = 21) are described as Pearson product-moment correlation coefficient (r). Results are statistically significant at P < 0.05. TPC: Total phenolic contents; TFC: Total flavonoid contents; RP: Reducing power.

### 2.8. Antimicrobial Activity

The antimicrobial activity of leaves, flowers and bark extracts varied significantly (P < 0.05) in relation to extracting solvents (Table 4, Table 5, Table 6). 80% methanol leaves extract was found to be the most effective, showing the lowest MIC (minimum inhibitory concentration) values (Table 4) against bacterial (*Pseudomonas stutzeri*, *Pseudomonas aeruginosa*, *Escherichia coli)* and fungal (*Aspergilus orazae*, *Aspergilus niger*, and *Fusarium solani)* strains. The positive controls (amoxicillin and flumequine) showed significantly lower MIC values against the tested bacteria and fungi. As expected, the negative controls showed no activity of the extracting solvents against any of the bacterial or fungal strains. 80% methanol flowers extract also showed appreciable antimicrobial activity against *P. stutzeri*, followed by *P. aeruginosa* and *E. coli* (Table 5). There was a uniform response of the deionized water extract against all organisms tested. In general, the antimicrobial activity of the tested leaves and flowers extracts (80% methanol) was comparable to that of the standard drugs amoxicillin and flumequine. Meanwhile, 80% methanol bark extract exhibited moderate activity against *P. aeruginosa*, *E. coli* and *A. niger.* On the other hand, acetone (both absolute and 80%) and water extracts from bark of Gold Mohar were found to be inactive against all organisms tested (Table 6). Gold Mohar has been widely used in several parts of the world for curing a wide range of ailments [4]. A varying antimicrobial activity of flowers, bark and pods extracts from this plant has been assessed previously [49,50,51].

Our report is the first for antimicrobial activity of leaves extract of this plant against the tested organisms. In this study, 80% methanol leaves and flowers extracts were found to be the most effective in inhibiting the growth of all the six tested microorganisms. The antimicrobial activity of leaves extracts against the tested microbes might be linked to the presence of phenolic acids such as gallic, protocatechuic, 3-hydroxybenzoic, chlorogenic acids and flavonoids [52]. Some researchers have reported that water and methanol extracts of pods and aqueous extracts of leaves of Gold Mohar did not show antimicrobial activity [50,51]. 

**Table 4:** Antimicrobial activity in terms of minimum inhibitory concentration of leaves extracts of Gold Mohar against the selected strains of bacteria and fungi.

Organism	Leaves extracts								
	Absolute	80%	Absolute	80%	Absolute	80%	Deionized	Amoxicillin	Flumequine
	Etanol	ethanol	methanol	methanol	acetone	acetone	water		
	Minimum inhibitory concentration (mg/mL)								
*Pseudomonas stutzeri*	25 ± 1.3 ^c^	22 ± 1.2 ^c^	21 ± 1.3 ^c^	20 ± 0.8 ^c^	65 ± 2.6 ^a^	50 ± 2.7 ^b^	70 ± 2.0 ^a^	21 ± 2.1 ^c^	-
*Pseudomonas aeruginosa*	35 ± 1.3 ^c^	30 ± 1.3 ^c^	26 ± 1.3 ^c^	23 ± 1.3 ^c^	75 ± 3.5 ^ab^	70 ± 3.3 ^b^	85 ± 1.8 ^a^	26 ± 2.4 ^c^	-
*Escherichia coli*	41 ± 2.1 ^c^	35 ± 2.4 ^c^	30 ± 2.7 ^c^	31 ± 1.4 ^c^	80 ± 3.2 ^b^	80 ± 3.1 ^b^	95 ± 2.3 ^a^	32 ± 1.8 ^c^	-
*Aspergilus orazae*	39 ± 2.1 ^b^	34 ± 2.3 ^bc^	32 ± 1.9 ^bc^	21 ± 1.9 ^c^	62 ± 3.2 ^a^	60 ± 4.1 ^a^	65 ± 3.4 ^a^	-	20 ± 1.1 ^c^
*Aspergilus niger*	45 ± 2.5 ^b^	40 ± 2.5 ^b^	35 ± 1.8 ^bc^	27 ± 1.1 ^c^	70 ± 4.2 ^a^	65 ± 4.4 ^a^	75 ± 3.3 ^a^	-	27 ± 1.2 ^c^
*Fusarium solani*	52 ± 3.3 ^b^	45 ± 3.1 ^bc^	42 ± 2.3 ^bc^	34 ± 1.2 ^c^	75 ± 4.3 ^a^	81 ± 2.8 ^a^	84 ± 2.9 ^a^	-	35 ± 1.3 ^c^

Values are mean ± SD of three separate experiments. Different superscript letters within the same row indicate significant (P < 0.05) differences of means within the extracting solvents.

**Table 5:** Antimicrobial activity in terms of minimum inhibitory concentration of flowers extracts of Gold Mohar against the selected strains of bacteria and fungi.

Organism	Flowers extracts								
	Absolute	80%	Absolute	80%	Absolute	80%	Deionized	Amoxicillin	Flumequine
	ethanol	ethanol	methanol	methanol	acetone	acetone	water		
	Minimum inhibitory concentration (mg/mL)								
*Pseudomonas stutzeri*	35 ± 1.8 ^c^	28 ± 0.9 ^c^	24 ± 1.1 ^d^	23 ± 1.3 ^d^	71 ± 3.3 ^a^	67 ± 2.6 ^ab^	65 ± 1.3 ^b^	21 ± 2.1 ^d^	-
*Pseudomonas aeruginosa*	45 ± 1.9 ^c^	41 ± 1.3 ^c^	35 ± 1.3 ^cd^	26 ± 1.1 ^d^	75 ± 3.4 ^a^	72 ± 3.3 ^a^	60 ± 1.7 ^b^	26 ± 2.4 ^d^	-
*Escherichia coli*	52 ± 2.0 ^bc^	50 ± 2.5 ^bc^	45 ± 2.3 ^c^	39 ± 1.1 ^cd^	88 ± 3.5 ^a^	83 ± 3.7 ^a^	66 ± 2.9 ^b^	32 ± 1.8 ^d^	-
*Aspergilus orazae*	56 ± 2.5 ^ab^	51 ± 1.3 ^b^	45 ± 1.2 ^c^	24 ± 1.1 ^cd^	70 ± 3.8 ^a^	68 ± 3.3 ^a^	68 ± 2.3 ^a^	-	20 ± 1.1 ^d^
*Aspergilus niger*	65 ± 2.8 ^ab^	57 ± 1.5 ^b^	49 ± 1.3 ^c^	30 ± 1.4 ^cd^	80 ± 2.9 ^a^	75 ± 2.3 ^a^	69 ± 2.7 ^ab^	-	27 ± 1.2 ^d^
*Fusarium solani*	70 ± 3.1 ^b^	65 ± 3.1 ^ab^	55 ± 2.8 ^c^	38 ± 1.8 ^d^	89 ± 3.4 ^a^	85 ± 2.8 ^a^	77 ± 2.9 ^ab^	-	35 ± 1.3 ^d^

Values are mean ± SD of three separate experiments. Different superscript letters within the same row indicate significant (P < 0.05) differences of means within the extracting solvents.

**Table 6:** Antimicrobial activity in terms of minimum inhibitory concentration of bark extracts of Gold Mohar against the selected strains of bacteria and fungi.

Organism	Bark extracts								
	Absolute	80%	Absolute	80%	Absolute	80%	Deionized	Amoxicillin	Flumequine
	ethanol	ethanol	methanol	methanol	acetone	acetone	water		
	Minimum inhibitory concentration (mg/mL)								
*Pseudomonas stutzeri*	60 ± 2.4 ^a^	55 ± 2.3 ^ab^	50 ± 2.3 ^ab^	45 ± 1.3 ^b^	-	-	-	21 ± 2.1 ^c^	-
*Pseudomonas aeruginosa*	65 ± 2.6 ^a^	60 ± 2.8 ^a^	55 ± 1.6 ^ab^	50 ± 1.3 ^b^	-	-	-	26 ± 2.4 ^c^	-
*Escherichia coli*	70 ± 3.8 ^a^	70 ± 2.9 ^a^	60 ± 3.5 ^b^	58 ± 1.8 ^b^	-	-	-	32 ± 1.8 ^c^	-
*Aspergilus orazae*	65 ± 3.1 ^a^	65 ± 2.1 ^a^	70 ± 3.1 ^a^	55 ± 2.4 ^b^	-	-	-	-	20 ± 1.1 ^c^
*Aspergilus niger*	75 ± 3.2 ^a^	70 ± 2.7 ^a^	65 ± 2.9 ^b^	60 ± 2.6 ^b^	-	-	-	-	27 ± 1.2 ^c^
*Fusarium solani*	80 ± 3.2 ^a^	75 ± 2.9 ^ab^	70 ± 3.3 ^ab^	65 ± 2.3 ^b^	-	-	-	-	35 ± 1.3 ^c^

Values are mean ± SD of three separate experiments. Different superscript letters within the same row indicate significant (P < 0.05) differences of means within the extracting solvents.

However, in agreement with our present findings, Aqil and Ahmad [21] reported a strong antimicrobial activity for the ethanolic (70%) extract of flowers of this plant. The significant difference (P < 0.05) in antimicrobial activity of the Gold Mohar extracts, with regard to different solvents, might be attributed to the variable nature and extracting ability of the solvents used. Some earlier reports showed that the changes in chemical composition of an extract directly affected their biological activities [15].

## 3. Experimental

### 3.1. Sample Collection and Preparation of Extracts

Three different samples of leaves, flowers and bark were collected from Gold Mohar [*Delonix regia* (Bojer ex Hook.) Raf.] plants grown in the vicinity of University of Agriculture Faisalabad (Faisalabad, Pakistan). The specimens were further identified and authenticated by Assistant Professor Mansoor Hameed, (Department of Botany, University of Agriculture, Faisalabad). Ambient-dried samples of leaves, flowers and bark were ground into a fine powder (80 mesh) in a grinding mill (Tector-Cemotec 1090 sample mill, Hognas, Sweden). For each of the dried parts (leaves, flowers and bark) material (20 g) was separately extracted with seven different solvents [absolute methanol, absolute ethanol, absolute acetone, 80% methanol (methanol:water, 80:20, v/v), 80% ethanol (ethanol:water, 80:20, v/v), 80% acetone (acetone:water, 80:20, v/v) and deionized water, 200 mL] as described earlier [39].

### 3.2. High Performance Liquid Chromatography (HPLC) Analysis

Identification of phenolic acids in the plant extract was performed on a Varian HPLC instrument using an ODS2 C_18_ reversed phase column [53]. The HPLC assays were conducted using acidified acetonitrile (99.5%) as mobile phase in isocratic mode with a constant flow rate of 1 mL/ min and detection at 280 nm. Sample injection volume was 20 µL. Phenolic compounds of each sample were identified by comparing their relative retention times with those of the standard mixture chromatogram. The concentration of an individual compound was calculated on the basis of peak area measurement and then converted to mg phenolics/100 g DW.

### 3.3. Determination of Total Phenolic Contents (TPC)

Amount of TPC was assessed using Folin-Ciocalteu reagent procedure as described earlier [54].

### 3.4. Determination of Total Flavonoid Contents (TFC)

The contents of total flavonoid were determined following the procedure described previously [55].

### 3.5. Antioxidant Activity Determination in Linoleic Acid System

The antioxidant activity of plant extracts was determined in terms of measurement of % inhibition of peroxidation in linoleic acid system following a method of Osawa and Namiki [56].

### 3.6. Determination of Reducing Power (RP)

The reducing power of the extract was determined according to the procedure described by Yen *et al*. [37].

### 3.7. DPPH Radical Scavenging Assay

The radical scavenging activity of the plant extracts against 2,2-diphenyl-1-picryl hydrazyl (DPPH^•^) radical was determined using a previously described method [57] with slight modifications. Aliquots of the extracts of various concentrations (10–100 µg/mL) were prepared in methanol. One milliliter of these extract concentrations was placed in test tubes and methanol (3 mL) was added followed by 1 mM methanol solution of DPPH^•^ (0.5 mL). A blank solution containing the same amount of methanol and DPPH^•^ was also prepared. After 30 min incubation at room temperature, the absorbance was read against blank at 517 nm. Inhibition of free radical by DPPH^•^ in percent (%) was calculated using following formula:

% inhibition of DPPH^•^ = {[Ab − Aa]/Ab} × 100

where Ab is the absorption of the blank sample and Aa is the absorption of the extract. IC_50_ values, which represented the extract concentration providing 50% inhibition of DPPH^•^ radicals, were calculated from the plot of inhibition percentage against extract concentration.

### 3.8. Antimicrobial Activity

The antimicrobial activity, *i.e.*, antibacterial and antifungal, of the extracts from leaves, flowers and bark of Gold Mohar was determined by measuring minimum inhibitory concentration [58] method. The pure bacterial and fungal strains were obtained from the National Institute for Biotechnology and Genetic Engineering (NIBGE), Faisalabad, Pakistan. Bacterial strains were cultured overnight at 37 °C in nutrient agar (Oxoid, Hampshire, UK), while fungal strains were cultured overnight at 30 °C using potato dextrose agar (Oxoid). For comparative purposes, amoxicillin and flumequine were used as positive controls for antibacterial and antifungal activities, respectively. Each of the solvents, in which extracts were prepared, was employed as negative control to discard the effect of solvents on microorganisms. The tube with least concentration of extract without growth after incubation was taken and recorded as the minimum inhibitory concentrations (MIC).

### 3.9. Statistical Analysis

Three different samples of leaves, flowers and bark were collected and analyzed individually in triplicate. The values are reported as mean ± SD of triplicate determinations (n = 3 × 3). The data were analyzed using one-way analysis of variance ANOVA using Minitab 2000 Version 13.2 statistical software (Minitab Inc., State College, PA, USA) at 5% significance level.

## 4. Conclusions

This study presents the first attempt to evaluate the differences in biological activities among leaves, flowers and bark of Gold Mohar. The results showed that extracts from leaves had higher antioxidant and antimicrobial activities compared to the extracts from flowers and bark, regardless of the solvent used. In addition, this study revealed that 80% methanol, had superior efficacy as an extracting solvent for recovering potent antioxidant components from Gold Mohar relative to other solvents, indicating an admirable potential of the related extracts for isolation of natural antioxidant and antimicrobial agents. HPLC analysis revealed that among the plant parts tested the leaves had relatively higher amounts of phenolic acids. Furthermore, the presented data would certainly help to ascertain the potency of the tested parts of Gold Mohar, especially the leaves, for medicinal health functions and functional food and nutraceutical applications.
